# Dynamic regulation of CD24 and the invasive, CD44^pos^CD24^neg ^phenotype in breast cancer cell lines

**DOI:** 10.1186/bcr2449

**Published:** 2009-11-11

**Authors:** Matthew J Meyer, Jodie M Fleming, Mustapha A Ali, Mitchell W Pesesky, Erika Ginsburg, Barbara K Vonderhaar

**Affiliations:** 1 Mammary Biology and Tumorigenesis Laboratory, Center for Cancer Research, National Cancer Institute, National Institutes of Health, 37 Convent Drive, Building 37, Room 1106, Bethesda, Maryland 20892-4254, USA

## Abstract

**Introduction:**

The invasive, mesenchymal phenotype of CD44^pos^CD24^neg ^breast cancer cells has made them a promising target for eliminating the metastatic capacity of primary tumors. It has been previously demonstrated that CD44^neg/low^CD24^pos ^breast cancer cells lack the ability to give rise to their invasive CD44^pos^CD24^neg ^counterpart. Here we demonstrate that noninvasive, epithelial-like CD44^pos^CD24^pos ^cells readily give rise to invasive, mesenchymal CD44^pos^CD24^neg ^progeny *in vivo *and *in vitro*. This interconversion was found to be dependent upon Activin/Nodal signaling.

**Methods:**

Breast cancer cell lines were sorted into CD44^pos^CD24^pos ^and CD44^pos^CD24^neg ^populations to evaluate their progeny for the expression of CD44, CD24, and markers of a mesenchymal phenotype. The populations, separated by fluorescence activated cell sorting (FACS) were injected into immunocompromised mice to evaluate their tumorigenicity and invasiveness of the resulting xenografts.

**Results:**

CD24 expression was dynamically regulated *in vitro *in all evaluated breast cancer cell lines. Furthermore, a single noninvasive, epithelial-like CD44^pos^CD24^pos ^cell had the ability to give rise to invasive, mesenchymal CD44^pos^CD24^neg ^progeny. Importantly, this interconversion occurred *in vivo *as CD44^pos^CD24^pos ^cells gave rise to xenografts with locally invasive borders as seen in xenografts initiated with CD44^pos^CD24^neg ^cells. Lastly, the ability of CD44^pos^CD24^pos ^cells to give rise to mesenchymal progeny, and *vice versa*, was blocked upon ablation of Activin/Nodal signaling.

**Conclusions:**

Our data demonstrate that the invasive, mesenchymal CD44^pos^CD24^neg ^phenotype is under dynamic control in breast cancer cell lines both *in vitro *and *in vivo*. Furthermore, our observations suggest that therapies targeting CD44^pos^CD24^neg ^tumor cells may have limited success in preventing primary tumor metastasis unless Activin/Nodal signaling is arrested.

## Introduction

The CD24 gene encodes a highly glycosylated, glycosylphosphatidylinositol anchored cell surface protein [1]. Thought to function as an adhesion molecule, it is known to bind Platelet Activation-Dependent Granule to External Membrane Protein (aka P-Selectin) [2] and facilitate intracellular signaling despite lacking a transmembrane domain [3]. In both normal and cancerous mammary tissue, CD24 positivity is frequently associated with a terminally differentiated, luminal phenotype [4-6]. In spite of this classification, the influence of CD24 expression on tumorigenicity and invasiveness is inconsistent, ranging from a positive [7-10] to a negative one [11-14].

Al-Hajj *et al*. [14] first described an impact of CD24 expression on breast cancer tumorigenicity by observing that CD44^pos^CD24^neg ^cells were highly tumorigenic in immunocompromised mice while CD44^pos^CD24^pos ^were nontumorigenic. Since then, the CD44/CD24 profile has been widely investigated in both primary tissues [4,15-22] and established breast cancer cell lines [13,23-31].

A relationship between CD24 and basal or luminal phenotype in breast cancer cell lines was reported by Fillmore and Kupperwasser [11]. Specifically, these authors demonstrated that cell lines with a high percentage of CD24^pos ^cells expressed luminal keratins while cell lines with a high percentage of CD24^neg ^cells expressed basal keratins. Consistent with these observations, CD44^high^CD24^neg ^cells were found to possess a basal/mesenchymal phenotype relative to CD44^low^CD24^pos ^cells [13]. Furthermore, using breast cancer cell lines, Sheridan *et al*. [27] demonstrated that CD44^pos^CD24^neg ^cells were more invasive than CD44^pos^CD24^pos ^cells. The invasive nature of CD44^pos^CD24^neg ^breast cancer cells has made this population a possible therapeutic target with the goal of eliminating the metastatic ability of primary tumors. Indeed, efforts to specifically target this population have been described [29-31].

Detailed comparisons between CD44^neg/low^CD24^pos ^and CD44^pos^CD24^neg ^breast cancer cells have been reported [4,13,32]. While CD44^neg/low^CD24^pos ^cells lack the ability to give rise to their invasive CD44^pos^CD24^neg ^counterpart [13], the regulation of CD24 and the invasive, CD44^pos^CD24^neg ^phenotype in CD44 positive breast cancer cells is less well understood. Our decision to work exclusively with CD44^pos ^cells was a deliberate effort to focus specifically on CD24 and avoid the well-described influence of CD44 expression on cell behavior [33-36].

Herein, we report that CD24 is under dynamic regulation *in vivo *and *in vitro *in five breast cancer cell lines. Specifically, CD44^pos^CD24^pos ^cells readily give rise to CD44^pos^CD24^neg ^cells and *vice versa*. Furthermore, noninvasive, epithelial-like CD44^pos^CD24^pos ^cells give rise to invasive, mesenchymal CD44^pos^CD24^neg ^progeny in an Activin/Nodal dependent manner. *In vivo*, this interconversion resulted in CD44^pos^CD24^pos ^cells giving rise to xenografts which had a similar capacity for local invasion as those initiated with CD44^pos^CD24^neg ^cells. These observations have potential clinical implications as specific targeting of CD44^pos^CD24^neg ^cells will leave behind CD44^pos^CD24^pos ^cells capable of giving rise to invasive progeny unless Activin/Nodal signaling is arrested.

## Materials and methods

### Cell culture

MCF7, ZR75.1, and MDA MB 231 cell lines were obtained from American Type Tissue Culture Collection (Manassas, VA, USA). MDA MB 231 and MCF7 cells were maintained in Dulbecco's Minimum Essential Medium (DMEM, Invitrogen, Gaithersburg, MD, USA) supplemented with 5% heat inactivated fetal bovine serum (FBS, Invitrogen), 10 μg/ml bovine insulin (Sigma, St. Louis, MO, USA), and 100 units/ml penicillin-streptomycin (Invitrogen). ZR75.1 cells were maintained in RPMI1640 (Invitrogen) supplemented with 10% heat inactivated FBS and 100 units/ml penicillin-streptomycin. MCF10Ca1a cells [37] (referred to as Ca1a, a kind gift of F.R. Miller, Wayne State University, Detroit, MI, USA, through L.M. Wakefield, CCR, NCI) were maintained in DMEM/F12 (Invitrogen) supplemented with 5% heat inactivated horse serum (HS, Gemini BioProducts, West Sacramento, CA, USA) and 100 units/ml penicillin-streptomycin. SUM159 cells (Asterand, Detroit, MI, USA) were maintained in Ham's F12 with 5% FBS, 5 μg/ml insulin, and 1 μg/ml hydrocortisone (Sigma). Cells were passaged following trypsinization (0.05% trypsin-EDTA, Invitrogen). The Activin/Nodal inhibitor SB-431542 [38,39] (Sigma) was solubilized in dimethyl sulfoxide (DMSO, Sigma) and supplemented to media at a final concentration of 10 μM and a final DMSO concentration of 0.1%. Cells not receiving SB-431542 were treated with 0.1% DMSO.

For generation of clonally derived cell lines, Ca1a cells were double-sorted and single cells plated directly into 96-well dishes containing conditioned DMEM/F12 media supplemented with 5% heat inactivated HS. Those wells containing a single cell were identified microscopically and expanded.

### Flow cytometric analysis and sorting

Anti-human CD44-allophycocyanin (APC, clone G44-26, 0.2 μg/ml final concentration) and anti-human CD24-phycoerythrin (PE, clone ML5, 26.6 μg/ml final concentration) or anti-human CD24-fluorescein (FITC, clone ML5, 26.6 μg/ml final concentration) (unless otherwise noted, all antibodies were purchased from BD Biosciences, Franklin Lakes, NJ, USA) were used for both analysis and live sorting. 7-aminoactinomycin D (7AAD, 1 μg/ml final concentration, BD Biosciences) was used for live/dead cell distinction. For flow cytometric analysis, cells were stained with a PBS solution containing 0.1% BSA and 0.1% sodium azide (Sigma) for 25 min at 4°C followed by two washes with this same buffer. For dual staining of CD24 and vimentin (PE, clone VI-RE/1, 10 μg/ml final concentration, Abcam, Cambridge, MA, USA) cells were stained with CD24-FITC as described above followed by fixation (0.1% formaldehyde, 15 min) and permeabilization (0.5% Tween 20, 10 min, Sigma). Staining was performed in a PBS solution containing 0.1% BSA, 0.1% sodium azide, and 0.5% Tween 20 for 25 min at 4°C followed by two washes with this same buffer. Analysis was performed on either a BD Biosciences FACSCalibur or LSR II. For dissociated xenografts, gates were established post-compensation with lineage^neg ^cells (devoid of anti-mouse CD45^neg ^[clone 30-F11, 6.7 ug/ml final concentration] and anti-mouse H-2Kd^neg ^[clone 15-5-5, 6.7 ug/ml final concentration] positive cells) that were not exposed to anti-human CD44 or anti-human CD24 antibodies.

For live sorting, cells were stained in a PBS solution containing 1.0% FBS, 100 units/ml penicillin-streptomycin, and 1 μg/ml Amphotericin B (Sigma) for 25 min at 4°C. Gates were established with unstained cells. Cell sorting was performed on a BD Biosciences FACSAria operating at Low Pressure (20 psi) using a 100 μm nozzle. Cell clusters and doublets were electronically gated out. Cells were routinely double-sorted and post-sort analysis typically indicated purities of > 90% with minimal cell death (< 10%). Flow cytometry data were analyzed using FlowJo v8.8.5 (TreeStar, Ashland, OR, USA).

### *In vivo *tumorigenicity and processing of xenografts

*In vivo *tumorigenicity was assessed by both frequency and latency of tumor formation in the abdominal mammary gland fat pad of 8 wk old athymic NCr-nu/nu mice obtained from the NCI colony (APA, Frederick, MD, USA). All animal experiments were conducted in accord with accepted standards of humane animal care and approved by the Animal Care and Use Committee at the National Institutes of Health. Five days prior to injection of cells, the bone marrow suppressant etoposide (VP-16) was administered intraperitoneally (ip, 30 mg/kg body weight, Calbiochem, Gibbstown, NJ, USA); animals also received a subcutaneous estrogen pellet (0.72 mg β-estradiol, 90-day release, Innovative Research of America, Sarasota, FL, USA). Cells were suspended in a F12 (Invitrogen)/Matrigel (high concentration, BD Biosciences) mixture (4:1) and injected into the mammary fat pad in a 50 μl volume. Mice were anesthetized by an ip injection of ketamine/xylazine (750 and 50 mg/kg body weight, respectively) in 200 μl Hank's Balanced Salt Solution (Invitrogen) prior to surgically exposing the gland for injection. Tumor size was measured weekly using a caliper. Experiments were terminated once a xenograft reached 1.0 cm in diameter or 75 d following injection of cells, whichever came first. Xenografts were removed, minced into < 1 mm pieces, and dissociated (F12 media containing 100 units/ml Collagenase type 3 (Worthington Biochemical Corp, Lakewood, NJ, USA), 0.8 units/ml Dispase (Invitrogen), and 100 units/ml penicillin-streptomycin) at 37°C under rotating conditions for 90 to 120 min. Single cells were generated by an additional incubation in 0.05% trypsin-EDTA for 5 min at 37°C. Hematoxylin and eosin (H&E) stained sections of mammary glands devoid of frank tumors were examined for the presence of macroscopic lesions.

### siRNA mediated knockdown of CD24

Non-targeting and CD24 siRNA pools were purchased from Dharmacon (Lafayette, CO, USA). Ca1a cells were transfected with 50 nM siRNA using DharmaFECT 1. Cells were harvested 72 hr post-transfection.

### Matrigel invasion assays

Cell invasion was assessed using Matrigel coated transwell chambers (8 μm, BD Biosciences). For analysis of sorted cells, cells were counted post-sorting using a Cellometer AutoT4 (Nexcelom Bioscience, Lawrence, MA, USA). For siRNA experiments, cells were trypsinized 24 hr post-transfection and counted. For both experiments, 30,000 cells were plated in triplicate in media containing 0.1% HS. Media containing 15% HS was used as the chemoattractant. Cells that had invaded 48 hr later were fixed with methanol, stained with 1% toluidine blue and counted under 20× magnification.

### Realtime RT-PCR

Total RNA was isolated from cells using the QIAGEN RNeasy kit (Valencia, CA, USA). The QIAGEN AllPrep DNA/RNA kit was used to isolate genomic DNA. RNA was reverse transcribed using Moloney murine leukemia virus reverse transcriptase (Invitrogen) primed with oligo-dT and random hexamers. The cDNA was then subjected to realtime PCR amplification using gene specific primers and 2× Brilliant II Sybr Green QPCR Mastermix (Roche Applied Science, Indianapolis, IN, USA). Primer sequences and PCR conditions are provided (see Additional data file 1). GADPH was employed as a housekeeping gene after confirming that it is expressed at similar levels between the CD44^pos^CD24^pos ^and CD44^pos^CD24^neg ^cells (see Additional data file 2). Data are presented as mean delta delta Ct relative to CD44^pos^CD24^pos ^cells.

### Immunoflouresence and confocal microscopy

Cells were either grown on ibidi 8-well chamber slides (Research Products International, Mt. Prospect, IL, USA) and fixed/permeabilized with ice cold acetone or sorted live, fixed/permeabilized with ice cold acetone followed by cytospin preparation. Following fixation, cells were blocked with 1% BSA. Primary antibodies (anti-Slug [clone D-19, 2 μg/ml final concentration] and anti-vimentin [clone H-84, 2 μg/ml final concentration], Santa Cruz Biotechnology, Santa Cruz, CA) were followed by the appropriate secondary antibody (anti-goat or anti-rabbit Alexa Fluor 594 or Alexa Fluor 488, 1:1000 dilution, Invitrogen). Imaging was performed using the Carl Zeiss LSM510 confocal imaging system (Carl Zeiss MicroImaging, Thornwood, NY, USA) at 63× magnification or an Olympus IX51 microscope (Olympus, Center Valley, PA, USA) at 20× magnification.

### Bisulfite sequencing

Bisulfite modification was performed on genomic DNA isolated from CD44^pos^CD24^pos ^or CD44^pos^CD24^neg ^sorted cells using the QIAGEN EpiTect Bisulfite Kit. Primers for PCR amplification were designed with MethPrimer [40] and a region spanning 366 bases and 28 CpG dinucleotides starting at -422 relative to the transcriptional start sight was queried (forward 5' GTTTATTAAATTGTTTAATGGTAATTA 3', reverse 5' ATCTTCCCAAAAACTAAAAAACC 3'). PCR products were cloned into DH5α cells by TOPO TA cloning (Invitrogen) and sequenced using M13 primers.

### RNA stability assay

Following sorting into CD44^pos^CD24^pos ^and CD44^pos^CD24^neg ^populations, cells were seeded into six-well dishes. One day later, cells were treated with 10 μg/ml Actinomycin-D (Sigma) and collected at 0, 4, 8, or 16 hr. RNA was isolated using Trizol (Invitrogen). Changes in CD24 mRNA were monitored by realtime RT-PCR.

### Statistics

Analysis of variance was performed using StatView 5.0.1 (SAS Institute, Cary, NC, USA). For analysis of realtime RT-PCR data, technical replicates for each gene from each of three independent experiments were averaged. Analysis of variance was performed on the resulting three independent values.

## Results

### CD24 expression is dynamically regulated in breast cancer cell lines

**Figure 1:**
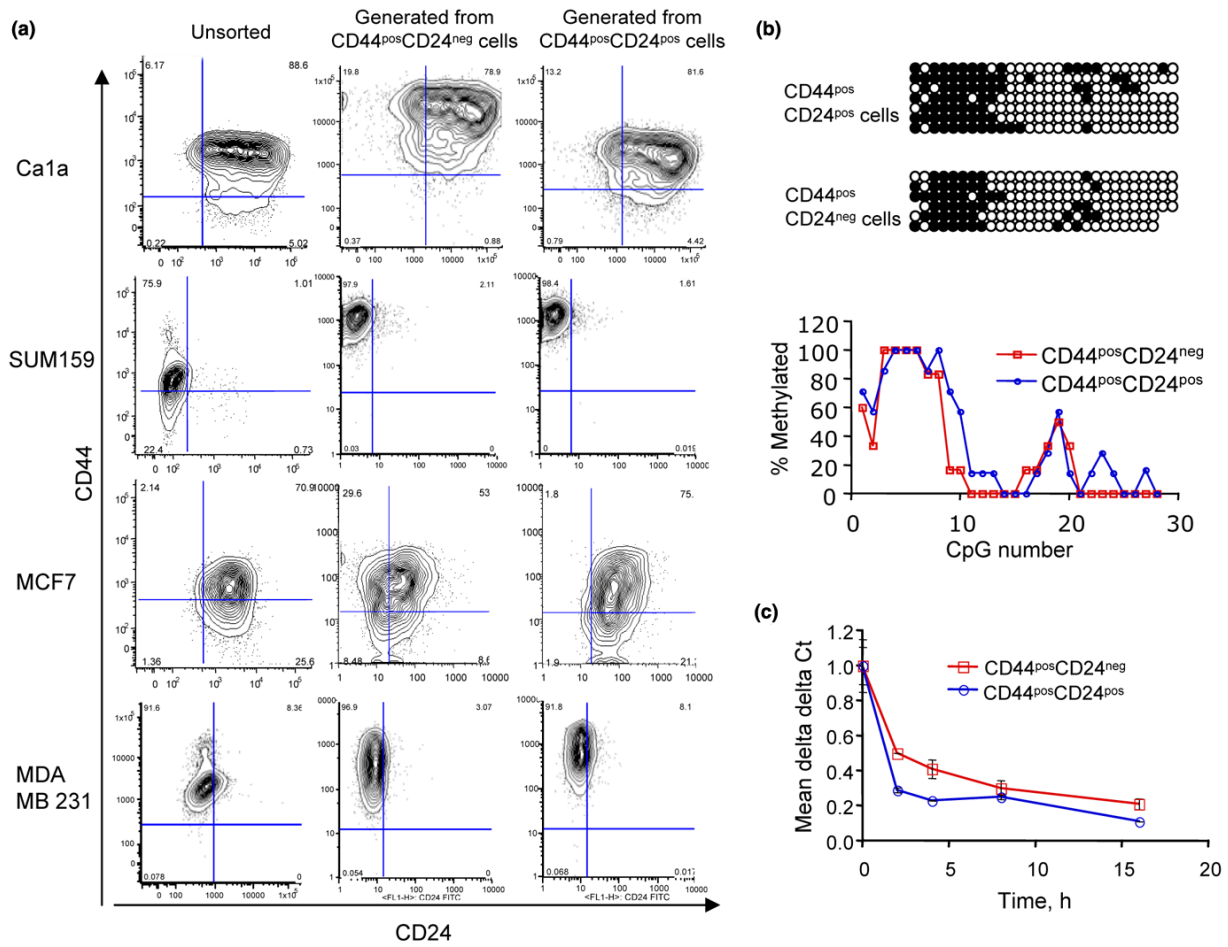
CD24 expression is dynamically regulated in breast cancer cell lines. (a) CD44^pos^CD24^neg ^and CD44^pos^CD24^pos ^cells were sorted from Ca1a, SUM159, MCF7, or MDA MB 231 breast cancer cell lines and expanded *in vitro*. The CD44, CD24 expression profile of the sorted cells was assessed via flow cytometry after two passages. (b) CD24 promoter CpG islands as predicted by MethPrimer (top panel). A 366 bp region was queried by bisulfate sequencing analysis (BS1, -422 to -788 relative to transcriptional start site). Bisulfite sequencing analysis of CD24 promoter in CD44^pos^CD24^pos ^and CD44^pos^CD24^neg ^parental Ca1a cells. Methylated CG (filled circles) and unmethylated CG (open circles) are presented (middle panel). Percentages of methylation of each CpG in the CD24 promoter from region queried (bottom panel). (c) CD24 mRNA stability assessed in Actinomycin treated, sorted cells. CD44^pos^CD24^pos ^(blue line) and CD44^pos^CD24^neg ^(red line) differences are presented as delta delta Ct means +/- standard deviations around the mean.

In an effort to understand the dynamics of CD24 expression in breast cancer cell lines, cells were sorted based on their CD44 CD24 expression and the CD44/CD24 expression of their progeny was evaluated. Nineteen breast cancer cells lines were initially screened for their expression of CD44 and CD24 (see Additional data file 3). Four cell lines (Ca1a, MCF7, SUM159 and MDA MB 231) were selected to evaluate the fluidity of CD24 expression *in vitro*. Cells were sorted into CD44^pos^CD24^neg ^and CD44^pos^CD24^pos ^populations (see Additional data file 4) and allowed to expand for two passages after which their CD44/CD24 expression was assessed by flow cytometry. For all four cell lines queried, CD44^pos^CD24^neg ^cells gave rise to CD44^pos^CD24^pos ^cells and *vice versa *(Figure 1a).

Data presented above suggests that CD24 expression is dynamically regulated in immortalized breast cancer cell lines. To evaluate if the CD24 gene was susceptible to dynamic transcriptional regulation, CpG methylation status of the CD24 promoter was queried in CD44^pos^CD24^neg ^and CD44^pos^CD24^pos ^populations sorted from the Ca1a cell line. A region spanning 366 bases (starting at -422 relative to the transcriptional start site) and 28 CpG dinucleotides was queried via bisulfite sequencing (Figure 1b). No differences in CpG methylation were observed between CD44^pos^CD24^neg ^and CD44^pos^CD24^pos ^cells. This suggests that rapid changes in CD24 transcription can occur without necessitating epigenetic modification of its promoter.

To further understand the regulation of CD24 expression, stability of the transcript was compared between CD44^pos^CD24^neg ^and CD44^pos^CD24^pos ^FACS sorted Ca1a cells. Following sorting, transcription was inhibited with Actinomycin-D and the rate of CD24 mRNA disappearance was evaluated. As indicated in Figure 1c, differences in CD24 abundance between CD44^pos^CD24^neg ^and CD44^pos^CD24^pos ^cells is not achieved by altered mRNA stability. CD24 expression as evaluated by flow cytometry could also be regulated at the translational level or by cell surface localization of the protein. However, given that cells devoid of the protein at the cell surface have markedly depressed levels of CD24 transcript (roughly one tenth that of CD24 positive cells) indicates that transcriptional regulation plays a considerable role in regulating CD24 protein expression.

### Noninvasive CD44^pos^CD24^pos ^cells give rise to invasive CD44^pos^CD24^neg ^cells

**Figure 2:**
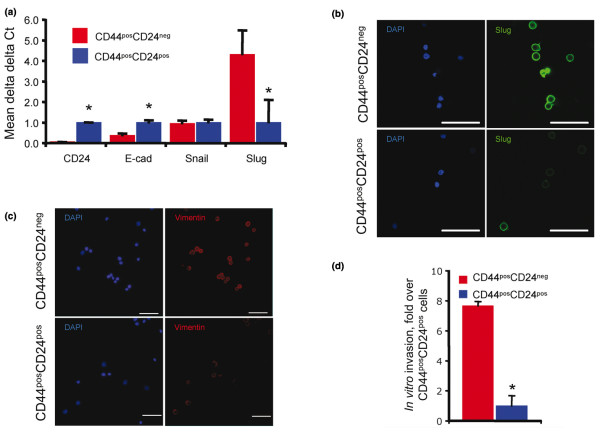
CD44^pos^CD24^neg ^cells possess an invasive, mesenchymal phenotype. (a) Total RNA was isolated from CD44^pos^CD24^neg ^and CD44^pos^CD24^pos ^cells sorted from parental Ca1a cells and transcript abundance was evaluated via realtime RT-PCR. CD44^pos^CD24^neg ^(red bars) and CD44^pos^CD24^pos ^(blue bars) differences are presented using the delta delta Ct method. Graphs represent means and associated standard errors of three independent experiments. * indicates *P *< 0.05. (b, c) Immunofluorescent staining of Slug (green) (b), vimentin (red) (c), and DAPI (blue) in cytospin preparations derived from parental Ca1a cells sorted into CD44^pos^CD24^neg ^and CD44^pos^CD24^pos ^populations. (d) Invasion through Matrigel by sorted parental Ca1a CD44^pos^CD24^neg ^(red bar) and CD44^pos^CD24^pos ^(blue bar) cells. Invaded cells were counted 48 hr following seeding. Graphs represent averages and associated standard errors of experiments performed in triplicate. * indicates *P *< 0.05.

We next set out to determine whether CD44^pos^CD24^pos ^cells could give rise to functional heterogeneity in addition to immunophenotypic heterogeneity as demonstrated above. It had been previously reported that CD44^pos^CD24^neg ^cells possess an invasive, mesenchymal phenotype relative to the epithelial-like phenotype of CD44^dim/pos^CD24^pos ^cells [13,27]. After sorting Ca1a cells, we confirmed that relative to CD44^pos^CD24^pos ^cells, the CD44^pos^CD24^neg ^population expressed elevated levels of Slug and vimentin and reduced levels of E-cadherin (Figure 2a, b, c). To confirm vimentin expression, Ca1a cells were dual stained for CD24 and vimentin. Consistent with data in Figure 2b, 92% of CD44^pos^CD24^neg ^cells were vimentin positive and expressed the protein at elevated levels (median fluorescence intensity = 1,494). While 32% of CD44^pos^CD24^pos ^cells fell in the vimentin positive gate, these cells expressed the protein at markedly lower levels (median fluorescence intensity = 7) than CD24^neg ^cells (see Additional data file 5). Furthermore, this population was nearly eight-fold more invasive through Matrigel than CD44^pos^CD24^pos ^cells (Figure 2d).

**Figure 3:**
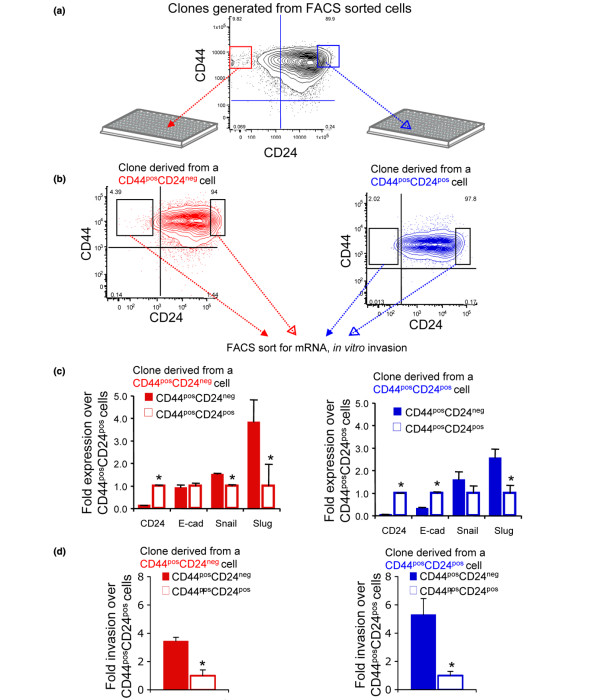
Clones generated from a single CD44^pos^CD24^pos ^or CD44^pos^CD24^neg ^cell possess molecular and functional heterogeneity similar to that of parental line. (a) Clones were generated by sorting single Ca1a CD44^pos^CD24^pos ^or CD44^pos^CD24^neg ^cells into individual wells of 96-well plates. Only wells confirmed to contain a single cell were expanded. (b) CD44/CD24 expression of representative clones derived from a single CD44^pos^CD24^neg ^or CD44^pos^CD24^pos ^cell. From the experiment described in (a), seven clones were generated from sorted CD44^pos^CD24^pos ^cells and five clones were generated from CD44^pos^CD24^neg ^cells. The CD44/CD24 profiles of representative clones are presented. (c) Total RNA was isolated from isogenic CD44^pos^CD24^neg ^and CD44^pos^CD24^pos ^cells sorted from the clones described in (a, b) and transcript abundance was evaluated via realtime RT-PCR. Data generated with cells sorted from a clone derived from a single CD44^pos^CD24^neg ^cell are presented in red. Data generated with cells sorted from a clone derived from a single CD44^pos^CD24^pos ^cell are presented in blue. CD44^pos^CD24^neg ^(closed bars) and CD44^pos^CD24^pos ^(open bars) differences are presented using the delta delta Ct method. Graphs represent means and associated standard errors of three independent experiments. * indicates *P *< 0.05. (d) Invasion through Matrigel by isogenic CD44^pos^CD24^neg ^and CD44^pos^CD24^pos^cells freshly sorted from the clones described in (a, b, c). 48 hr following seeding, invaded cells were counted. Graphs represent averages and associated standard errors of experiments performed in triplicate. Data generated with cells sorted from a clone derived from a single CD44^pos^CD24^neg ^cell are presented in red. Data generated with cells sorted from clone derived from a single CD44^pos^CD24^pos ^cell are presented in blue. * indicates *P *< 0.05.

We took advantage of these differences between CD44^pos^CD24^pos ^and CD44^pos^CD24^neg ^cells to evaluate if either population possessed the ability to give rise to molecular and functional heterogeneity. Specifically, we set out to determine if the CD44^pos^CD24^neg ^progeny of noninvasive CD44^pos^CD24^pos ^cells possessed an invasive, mesenchymal phenotype. To address this question in the most stringent manner possible, clones were propagated from CD44^pos^CD24^neg ^or CD44^pos^CD24^pos ^Ca1a cells (Figure 3a). Following a double sort, single cells were deposited into 96-well dishes and expanded. Only wells confirmed to contain a single cell after sorting (determined microscopically) were evaluated. Less than 1.5% of CD44^neg ^cells were able to generate clones, independent of CD24 status, indicating that these cells lack self-renewal properties (data not shown). Seven clones were generated from sorted CD44^pos^CD24^pos ^cells and five clones were generated from CD44^pos^CD24^neg ^cells with roughly 30% of single cells giving rise to a successful colony, independent of CD24 expression (data not shown). For all clones, CD44^pos^CD24^neg ^cells gave rise to CD44^pos^CD24^neg ^cells, and *vice versa *(see Additional data file 6). FACS profiles of clones derived from a CD44^pos^CD24^pos ^cell or a CD44^pos^CD24^neg ^cell are presented in Figure 3b demonstrating the ability of a single CD44^pos^CD24^pos ^cell to give rise to isogenic CD44^pos^CD24^neg ^progeny, and *vice versa*. These observations confirmed data generated with bulk sorted Ca1a, SUM159, MCF7, and MDA MB 231 cells (Figure 1a).

As presented in Figure 2, the parental Ca1a cell line possesses two functionally unique populations (invasive CD44^pos^CD24^neg ^cells and noninvasive CD44^pos^CD24^pos ^cells). To determine if either CD44^pos^CD24^pos ^or CD44^pos^CD24^neg ^cells possessed the ability to give rise to this molecular and functional heterogeneity, the clones described above were sorted and queried for expression of mesenchyme-related genes as well as invasiveness through Matrigel. We observed that a single noninvasive, epithelial-like CD44^pos^CD24^pos ^cell had the ability to give rise to isogenic, CD44^pos^CD24^neg^progeny possessing elevated levels of Snail and Slug and reduced levels of E-cadherin (Figure 3c). Furthermore, these CD44^pos^CD24^neg ^progeny were 5-fold more invasive than their CD44^pos^CD24^pos ^parental cell (Figure 3d). Likewise, a single CD44^pos^CD24^neg ^cell had the ability to give rise to noninvasive, epithelial-like, CD44^pos^CD24^pos ^progeny (Figures 3c, d). These data demonstrate that CD44^pos^CD24^pos ^cells are plastic and can readily give rise to progeny possessing molecular and functional characteristics unlike their own.

### Xenografts derived from CD44^pos^CD24^pos ^cells are locally invasive and contain CD44^pos^CD24^neg ^progeny

**Figure 4:**
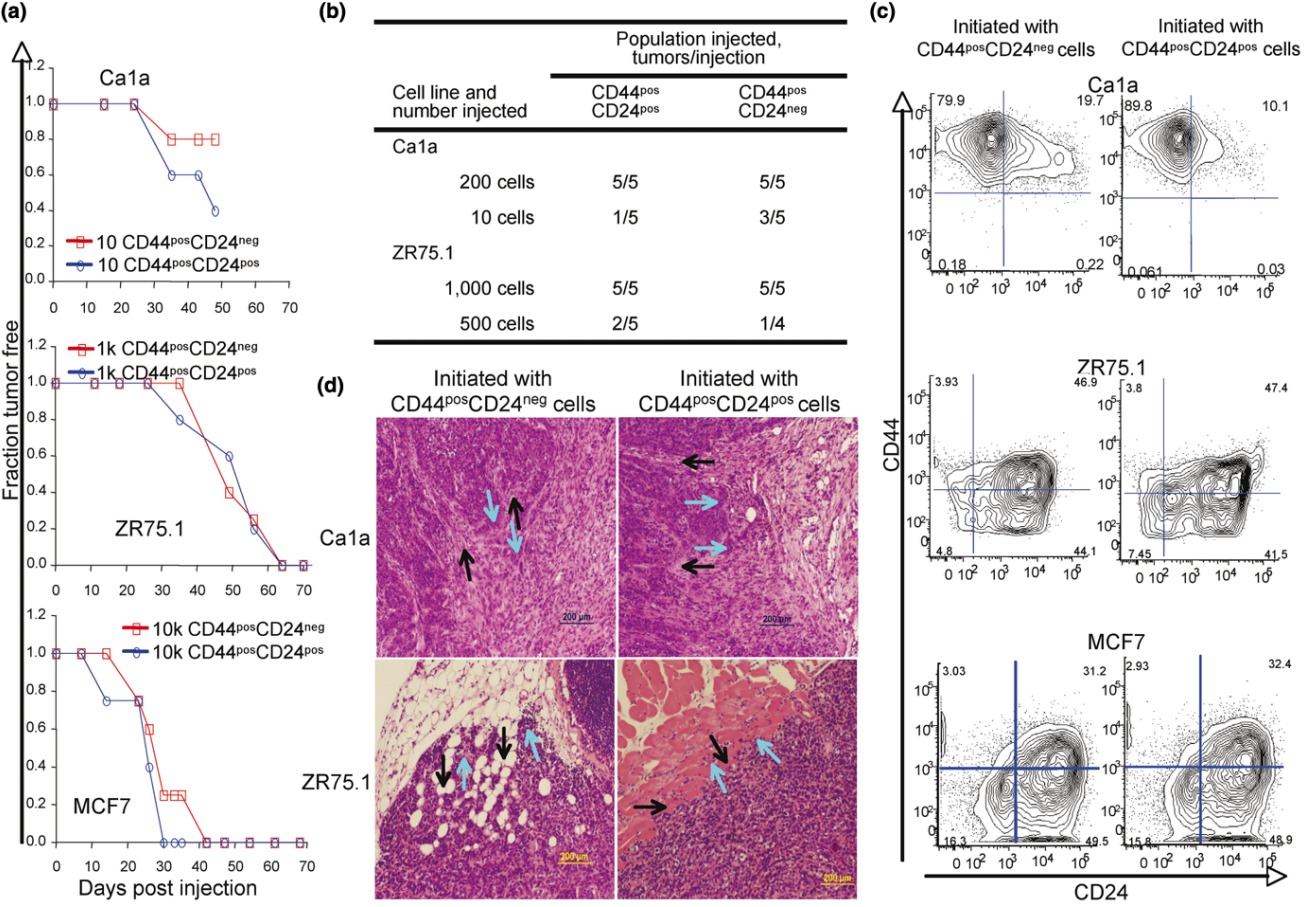
Xenografts derived from CD44^pos^CD24^pos ^cells are locally invasive and contain CD44^pos^CD24^neg ^progeny. (a) Ca1a (top), ZR75.1 (middle), and MCF7 (bottom) breast cancer cells were FACS sorted based on their CD44, CD24 expression and injected into the abdominal mammary fat pad of nu/nu mice in 50 μl of a 25% Matrigel suspension. Latency of tumor formation following orthotopic injection of sorted CD44^pos^CD24^neg/dim ^(red line) or CD44^pos^CD24^pos ^(blue line) breast cancer cells is presented. Five mice per population were injected and palpated weekly. (b) Limiting dilution of tumor initiating cells in sorted ZR75.1 and Ca1a cells. (c) Xenografts established with either CD44^pos^CD24^neg ^or CD44^pos^CD24^pos ^cells were dissociated and evaluated for CD44/C24 expression. Host cells were excluded with mouse anti-CD45, and -H-2K^d ^antibodies as described in the Methods section. Representative FACS profiles of xenografts initiated with sorted Ca1a, ZR75.1, and MCF7 cells are presented. (d) Local invasion of xenografts established by injecting CD44^pos^CD24^neg ^or CD44^pos^CD24^pos ^sorted Ca1a or ZR75.1 cells into the abdominal mammary fat pad of nude mice. Representative H&E stained sections of the resulting xenografts are presented. Black arrows indicate host tissue. Light blue arrows indicate invading tumor cells.

Data presented above demonstrate that noninvasive CD44^pos^CD24^pos ^cells readily give rise to invasive CD44^pos^CD24^neg ^progeny. To determine if this is limited to *in vitro *conditions, three cell lines (Ca1a, MCF7 and ZR75.1) were sorted into CD44^pos^CD24^pos ^and CD44^pos^CD24^neg ^populations and injected into the abdominal fat pad of immunocompromised mice. Not surprisingly, we observed differences among cell lines in tumorigenicity (Figure 4a, b). In all cases, within cell lines, CD44^pos^CD24^pos ^and CD44^pos^CD24^neg ^populations were equally tumorigenic (Figure 4a, b). In the case of Ca1a, 10 cells from either CD44^pos^CD24^pos ^or CD44^pos^CD24^neg ^cells resulted in a similar frequency (Figure 4a) of equally sized tumors (*P *= 0.89, data not shown). Both populations gave rise to tumors greater than 1 cm in diameter within 50 days of injection. One thousand ZR75.1 cells, independent of CD24 status, resulted in 100% of mice developing tumors by 62 days post injection (Figure 4a) with CD44^pos^CD24^neg ^cells yielding 1.9 fold larger tumors than CD44^pos^CD24^pos ^cells (*P *< 0.05, data not shown). When 500 ZR75.1 cells were injected, 2/5 mice and 1/4 mice developed tumors by 75 days when injected with CD44^pos^CD24^pos ^or CD44^pos^CD24^neg ^cells, respectively (Figure 4b) without a difference in tumor volume (*P *= 0.56, data not shown). Likewise, the injection of 10,000 MCF7 cells resulted in 100% tumor incidence within 50 days (Figure 4a) with no difference in tumor volume (*P *= 0.23, data not shown).

Once xenografts reached 1 cm in diameter they were removed, dissociated, and subjected to flow cytometric analysis. Contaminating host cells were excluded by gating out H-2K^d pos ^and mouse specific CD45^pos ^cells. While the CD44/CD24 profile of resulting xenografts is not identical to that of the parental cell line, CD44^pos^CD24^pos ^cells readily gave rise to CD44^pos^CD24^neg ^progeny *in vivo*, and *vice versa *(Figure 4c). This latter observation is consistent with our *in vitro *observations. More importantly, we observed that xenografts initiated with either CD44^pos^CD24^pos ^or CD44^pos^CD24^neg ^cells had a capacity for local invasion (Figure 4d). These observations confirmed that progeny of noninvasive CD44^pos^CD24^pos ^cells yield progeny capable of invading surrounding tissues.

### Requirement for Activin/Nodal signaling in the generation of molecular heterogeneity

**Figure 5:**
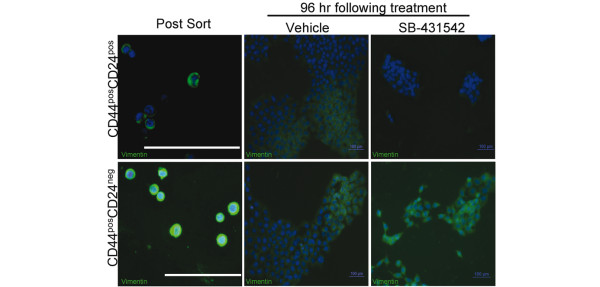
Role of Activin/Nodal signaling in the generation of molecular heterogeneity. Ca1a cells were sorted into CD44^pos^CD24^neg ^and CD44^pos^CD24^pos ^populations. Cytospin prepared cells were stained for vimentin immediately following sorting (left two panels, bar represents 200 μm). Sorted cells were allowed to expand for 96 hr in the presence of vehicle (0.1% DMSO, middle two panels) or 10 μM SB-431542 (right two panels). Immunofluorescent staining of vimentin (green) and DAPI (blue) is presented. In the absence of drug, CD44^pos^CD24^pos^cells yield mesenchymal, vimentin positive progeny. Inhibition of Activin/Nodal signaling prevents this interconverstion.

The role Activin/Nodal signaling plays in the generation of molecular and functional heterogeneity by CD44^pos^CD24^pos ^and CD44^pos^CD24^neg ^cells was explored with the use of SB-431542, a small molecule inhibitor of ALK4, -5, -7 [38,39]. Immediately post-sorting, vimentin expression was greatest in CD44^pos^CD24^neg ^cells and low/negative in CD44^pos^CD24^pos ^subpopulations (Figures 2c, 5). As expected, 96 hours post-sorting, vehicle treated CD44^pos^CD24^pos ^cells and CD44^pos^CD24^neg ^cells gave rise to progeny with molecular heterogeneity (Figure 5). Specifically, epithelial-like, vimentin negative/low CD44^pos^CD24^pos ^cells gave rise to mixed progeny; some expressed high levels of vimentin and others lacked the mesenchymal marker. Similarly, mesenchymal, vimentin positive CD44^pos^CD24^neg ^cells expanded giving rise to a mixed population of vimentin negative and positive progeny. Following treatment with SB-431542, however, vimentin low/negative CD44^pos^CD24^pos ^cells gave rise to uniformly vimentin negative progeny. CD44^pos^CD24^neg ^cells treated with SB-431542 gave rise to homogeneously vimentin positive progeny (Figure 5). These data demonstrate that active Activin/Nodal signaling is not required for expansion of either CD44^pos^CD24^pos ^or CD44^pos^CD24^neg ^cells. However, both populations require this pathway in order to give rise to molecular heterogeneity. Specifically, Activin/Nodal signaling is required for vimentin positive, CD44^pos^CD24^neg ^cells to give rise to vimentin negative progeny and for vimentin negative, CD44^pos^CD24^pos ^cells to give rise to vimentin positive progeny.

### Depletion of CD24 caused increased invasiveness without yielding a mesenchymal phenotype

**Figure 6:**
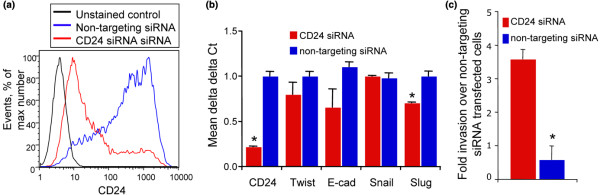
Depletion of CD24 caused increased invasiveness without yielding a mesenchymal phenotype. (a) Ca1a cells were transfected with non-targeting or CD24 siRNA. Histogram illustrates CD24-PE fluorescence intensity of unstained cells (black line) and cells transfected with CD24 siRNA (red line) or non-targeting siRNA (blue line) as assessed by flow cytometric analysis. (b) Total RNA was isolated from cells following transfection with non-targeting or CD24-targeting siRNA. Transcript abundance was evaluated via realtime RT-PCR. Non-targeting siRNA transfected cell (blue bars) and CD24-targeting siRNA transfected cell (red bars) differences are presented using the delta delta Ct method. Graphs represent means and associated standard errors of experiments performed in triplicate. * indicates *P *< 0.05. (c), 24 hr following transfection with either non-targeting or CD24 siRNA, Ca1a cells were trypsinized, counted, and seeded to Matrigel invasion chambers in triplicate. Invaded cells were counted 48 hr later. Graph represents mean fold change in invaded cells associated with CD24 siRNA transfection and associated standard errors. * indicates *P *< 0.05.

We next sought to evaluate whether the lack of CD24 expression is upstream or downstream of the mesenchymal phenotype associated with CD24 negativity. Seventy two hours following transient transfection using a pool of siRNA targeting CD24 yielded a seven-fold increase in the percentage of CD24^neg ^cells and a concomitant 26-fold decrease in median fluorescence intensity relative to cells transfected with non-targeting siRNA (Figure 6a). Depletion of CD24 expression did not yield a mesenchymal phenotype based on the expression of E-cadherin, Snail, Slug, and Twist (Figure 6b) but instead resulted in a reduction in Slug mRNA (*P *< 0.05). Consistent with an apparent lack of epithelial to mesenchymal transition, CD24 siRNA similarly failed to alter cell morphology (data not shown). Despite this lack of mesenchymal phenotype, CD24 siRNA transfected cells were 3.5-fold more invasive than non-targeting siRNA transfected cells (Figure 6c). In the invasion experiments, cells were counted and seeded to invasion chambers 24 h post transfection. The number of invading cells was counted 72 h post transfection. These data indicate that exogenous down regulation of CD24 is sufficient to yield increased invasiveness. However, it is unable to elicit a mesenchymal phenotype associated with endogenous down regulation of CD24.

## Discussion

Herein, we demonstrate that noninvasive, epithelial-like CD44^pos^CD24^pos ^cells readily give rise to invasive, mesenchymal CD44^pos^CD24^neg ^progeny. This plasticity, which is dependent upon Activin/Nodal signaling, is the likely mechanism by which noninvasive, epithelial-like CD44^pos^CD24^pos ^cells give rise to xenografts with locally invasive boundaries.

Cell motility is a fundamental aspect to early cancer metastasis. The ability of single cells to move from the primary tumor is frequently facilitated via the transition from an epithelial to a mesenchymal phenotype. Indeed, tumors that possess a mesenchymal gene signature correlate with tumor progression and poor prognosis [41-43]. As such, direct targeting of the invasive, mesenchymal component of primary breast cancer could be of substantial clinical benefit. The acquisition of a mesenchymal phenotype is associated with, among other things, the loss of E-cadherin [44] and increased vimentin expression [45]. Recently, CD44^pos^CD24^neg ^breast cancer cells were demonstrated to possess this mesenchymal phenotype [13] and we herein extended these observations. The specific targeting of CD44^pos^CD24^neg ^cells has proven effective at reducing the frequency of this population [29-31]. Our interest was in broadening the understanding of regulation of the CD24 gene and the invasive, mesenchymal CD44^pos^CD24^neg ^population in breast cancer cell lines.

Molecular and functional differences between CD44^neg/dim^CD24^pos ^and CD44^pos^CD24^neg ^cells have been eloquently described, including the observation that the former cannot give rise to the latter [4,13,32]. However, CD44 expression is known to profoundly impact cell behavior. Relative to CD44^pos ^cancer cells, those with low to no CD44 expression have reduced growth, invasiveness, and tumorigenicity, heightened susceptibility to chemotherapeutics, and reduced levels of pluripotent stem cell markers [33,34,46-48]. Indeed, we observed that fewer than 2% of CD44^dim/neg ^cells (independent of CD24 status) gave rise to colonies *in vitro*. Due to the well-characterized dominant effect of CD44 on cell behavior and the fact that previous work has compared CD44^dim/neg ^to CD44^pos ^cells [4,13,32], the regulation of CD24 and its specific role in breast cancer cell behavior is largely unknown.

We demonstrated *in vitro *and *in vivo *that CD24 expression is dynamically regulated. Specifically, CD44^pos^CD24^pos ^cells readily gave rise to CD44^pos^CD24^neg ^progeny and *vice versa*. This was stringently confirmed *in vitro *by demonstrating that clones derived from a single CD44^pos^CD24^pos ^cell yielded CD44^pos^CD24^neg ^progeny. In non-transformed mammary epithelial cells, CD24 positivity is frequently associated with a terminally differentiated, luminal phenotype [5,6,49]. Such lineage commitment and long-term modification of gene expression is frequently achieved via alterations in promoter CpG dinucleotide methylation [50,51]. In our study, bisulfite sequencing analysis revealed that CD24 promoter methylation is similar between CD44^pos^CD24^neg ^and CD44^pos^CD24^pos ^cells suggesting that transcription can be rapidly altered without requiring changes in promoter methylation. Data presented herein do not rule out regulation of CD24 expression by modified translation or cell surface localization of the protein. However, these findings are consistent with our data demonstrating that the gene is indeed susceptible to dynamic transcriptional regulation. Furthermore, others have shown in MCF10A, a normal mammary cell line, that CD24 expression is under the regulatory control of Wnt signaling [52].

More importantly, the clones we generated confirmed that CD44^pos^CD24^pos ^cells give rise to functionally heterogeneous progeny. Specifically, we demonstrated that a single noninvasive, epithelial-like CD44^pos^CD24^pos ^cell could give rise to CD44^pos^CD24^neg ^progeny with an invasive, mesenchymal phenotype. Similarly, xenografts initiated with CD44^pos^CD24^pos ^cells contained CD44^pos^CD24^neg ^progeny. Furthermore, these xenografts were as invasive as those initiated with CD44^pos^CD24^neg ^cells. These observations demonstrate that while CD44^pos^CD24^pos ^cells are noninvasive, they are fully capable of giving rise to invasive progeny.

Recently, Chang *et al*. [53] described a similar phenomenon in clones derived from Sca-1^high ^and Sca-1^low ^multipotent mouse hematopoietic cells. They reported that isogenic Sca-1^high ^and Sca-1^low ^cells, despite both being multipotent, had divergent global gene expression profiles and were functionally different. Furthermore, Sca-1^high ^cells gave rise to Sca-1^low ^cells and *vice versa*. Our findings, and those of Chang *et al*. [53], demonstrate the fundamental plasticity in functional heterogeneity present in isogenic mammalian cells.

Efforts are currently underway to specifically target CD44^pos^CD24^neg ^breast cancer cells due to their invasive, mesenchymal phenotype [29-31] and hypothesized role in seeding distant metastases. The data described herein have potential clinical implications as specific targeting of CD44^pos^CD24^neg ^cells will leave behind CD44^pos^CD24^pos ^cells that we demonstrate are capable of giving rise to invasive progeny. In an effort to address this, we sought to identify key pathways required by CD44^pos^CD24^pos ^cells to give rise to mesenchymal progeny. Relative to CD44^neg^CD24^pos ^breast cancer cells, Shipitsin *et al*. [4] found the TGFβ pathway was active in CD44^pos^CD24^neg ^cells. CD44 expression has been demonstrated to regulate TGFβ signaling [35,54], so we chose to evaluate the influence of CD24 expression on Activin/Nodal signaling and *vice versa *in CD44^pos ^cells. To do so, we treated CD44^pos^CD24^neg ^and CD44^pos^CD24^pos ^cells with the Activin/Nodal inhibitor, SB-431542 [38,39]. These experiments demonstrated that Activin/Nodal signaling was not required for the expansion of either population, *i.e*. vimentin negative CD44^pos^CD24^pos ^cells expanded giving rise to vimentin negative progeny in the presence of the drug. Likewise, SB-431542 treated vimentin positive CD44^pos^CD24^neg ^cells gave rise to vimentin positive progeny. However, we demonstrated that both CD44^pos^CD24^pos ^and CD44^pos^CD24^neg ^cells require Activin/Nodal signaling in the generation of phenotypically diverse progeny. Most substantially, SB-431542 exposure to epithelial-like CD44^pos^CD24^pos ^cells blocked their ability to give rise to mesenchymal, vimentin positive progeny. These findings also demonstrate that despite the molecular and functional differences between CD44^pos^CD24^pos ^and CD44^pos^CD24^neg ^cells, both populations share a similar requirement for Activin/Nodal signaling in the generation of functionally heterogeneous progeny, thus making this pathway an exciting candidate to target clinically.

**Figure 7:**
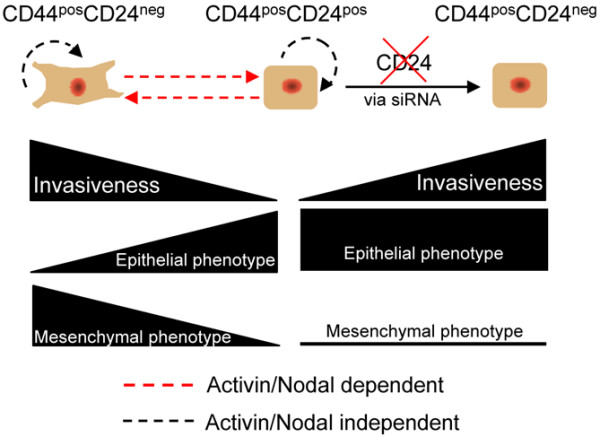
Dynamic regulation of CD24 and the invasive CD44^pos^CD24^neg ^phenotype. Schematic representation of the plasticity of the CD44^pos^CD24^neg^, invasive/mesenchymal phenotype. Inhibition of Activin/Nodal signaling blocks the ability of CD44^pos^CD24^pos ^cells to give rise to invasive, mesenchymal progeny and CD44^pos^CD24^neg ^cells are prevented from giving rise to non-invasive, epithelial progeny. Depletion of CD24 via siRNA causes increased invasiveness without yielding a mesenchymal phenotype.

When CD24 expression was depleted exogenously, cell invasiveness increased. However, this invasiveness was not associated with changes in gene expression seen when CD24 expression is reduced endogenously. Increased invasiveness in the absence of elevated Snail or Slug expression has been previously reported in the literature. Specifically, β-catenin-lymphoid enhancer factor-1 expression yields increased invasiveness in colon carcinoma without increasing Snail or Slug expression [55]. Our observations suggest that the endogenous down regulation of CD24 is likely not an upstream event in the acquisition of the invasive, mesenchymal phenotype by CD44^pos^CD24^neg ^progeny of CD44^pos^CD24^pos ^cells. However, the current experiments were not able to determine if exogenous depletion of CD24 yielded a phenotype with similar levels of invasiveness as cells devoid of CD24 via endogenous means. A diagram outlining the proposed role of Activin/Nodal signaling in the regulation of CD24 and the invasive CD44^pos^CD24^neg ^phenotype is provided in Figure 7.

## Conclusions

Herein we report that while CD44^pos^CD24^pos ^breast cancer cells represent a noninvasive, epithelial phenotype, they give rise to xenografts with a profound capacity for local invasion. This ability to form invasive tumors was ascribed to the fact that CD44^pos^CD24^pos ^cells readily give rise to CD44^pos^CD24^neg ^cells that possess an invasive, mesenchymal phenotype. The plasticity of CD44^pos^CD24^pos ^cells was blocked with SB-431542 indicating that ablation of Activin/Nodal signaling may be required in combination with therapies targeting CD44^pos^CD24^neg ^cells when breast cancer cell lines are used as models.

## Abbreviations

7AAD: 7-aminoactinomycin D; APC: allophycocyanin; E-cad: E-cadherin; FACS: fluorescence activated cell sorting; FBS: fetal bovine calf serum; FITC: fluorescein; neg: negative; PE: phycoerythrin; pos: positive;

## Competing interests

The authors declare that they have no competing interests.

## Authors' contributions

MJM developed ideas, conceived the experiments and wrote and edited the manuscript. MJM, JMF, MAA, MP and EG conducted the experiments. JMF and EG edited the manuscript. BKV developed ideas and edited the manuscript. All authors contributed to the analysis of data.

## Supplementary Material

Additional file 1 A table containing realtime PCR primer sequences and conditions. Click here for file

Additional file 2 A table containing GADPH Ct values for CD44^pos^CD24^neg ^and CD44^pos^CD24^pos ^cells. Click here for file

Additional data file 3 A table containing the estrogen receptor, progesterone receptor, HER2 amplification, and CD44/CD24 expression in 19 breast cancer cell lines. Click here for file

Additional data file 4 A table containing the CD44/CD24 expression profile of clones derived from a single CD44^pos^CD24^pos ^or CD44^pos^CD24^neg ^cell. Click here for file

Additional file 5 A figure containing representative post sort analyses of sorted Ca1a, SUM 159 and MCF7 cells. Click here for file

Additional file 6 A figure containing representative flow cytometric quantitation of vimentin expression by CD44^pos^CD24^pos ^and CD44^pos^CD24^neg ^Ca1a cells. Click here for file
