# Supplementary material for: Dynamic regulation of CD24 and the invasive, CD44posCD24neg phenotype in breast cancer cell lines
Source: Breast Cancer Res. 2009 Nov 11;11(6):R82. doi: 10.1186/bcr2449 (PMC2815544; doi:10.1186/bcr2449)
Supplement: Additional file 1 — A table containing realtime PCR primer sequences and conditions. [file bcr2449-S1.DOC]

**Additional Data File 1.** Realtime PCR primer sequences and conditions

| Gene | Forward primer | Reverse primer | Tm, ˚C |
| --- | --- | --- | --- |
| CD24 | CCCACGCAGATTTATTCCAG | GACTTCCAGACGCCATTTG | 57 |
| Twist1 | GGAGTCCGCAGTCTTACGAG | TCTGGAGGACCTGGTAGAGG | 57 |
| Snail1 | CGAAAGGCCTTCAACTGCAAAT | ACTGGTACTTCTTGACATCTG | 57 |
| Slug | TGATGAAGAGGAAAGACTACAG | GCTCACATATTCCTTGTCACAG | 57 |
| E-cadherin | ATTCTGATTCTGCTGCTCTTG | AGTAGTCATAGTCCTGGTCTT | 57 |
| GADPH | TGATGACATCAAGAAGGTGG | TCTTACTCCTTGGAGGCC | 57 |
