# Supplementary material for: Dynamic regulation of CD24 and the invasive, CD44posCD24neg phenotype in breast cancer cell lines
Source: Breast Cancer Res. 2009 Nov 11;11(6):R82. doi: 10.1186/bcr2449 (PMC2815544; doi:10.1186/bcr2449)
Supplement: Additional file 2 — A table containing GADPH Ct values for CD44posCD24neg and CD44posCD24pos cells. [file bcr2449-S2.DOC]

**Additional Data File 2. GADPH Ct values for CD44^pos^CD24^neg^ and CD44^pos^CD24^pos^ cells.**

| Cell Line/ Experiment | Sorted Population/  GADPH Ct | |
| --- | --- | --- |
|  | CD44^pos^CD24^pos^ | CD44^pos^CD24^neg^ |
| Parental |  |  |
| Exp 1 | 15.86 | 15.66 |
| Exp 2 | 16.88 | 16.93 |
| Exp 3 | 14.02 | 13.89 |
| CD44^pos^CD24^pos^ clone | |  |
| Exp 1 | 17.88 | 16.46 |
| Exp 2 | 18.12 | 17.95 |
| Exp 3 | 17.06 | 17.37 |
| CD44^pos^CD24^neg^ clone | |  |
| Exp 1 | 16.23 | 16.46 |
| Exp 2 | 13.89 | 13.86 |
| Exp 3 | 17.37 | 17.26 |
