# Supplementary material for: Dynamic regulation of CD24 and the invasive, CD44posCD24neg phenotype in breast cancer cell lines
Source: Breast Cancer Res. 2009 Nov 11;11(6):R82. doi: 10.1186/bcr2449 (PMC2815544; doi:10.1186/bcr2449)
Supplement: Additional data file 3 — A table containing the estrogen receptor, progesterone receptor, HER2 amplification, and CD44/CD24 expression in 19 breast cancer cell lines. [file bcr2449-S3.DOC]

**Additional Data File 3**. Estrogen receptor, progesterone receptor, HER2 amplification, and CD44/CD24 expression in 19 breast cancer cell lines.

| Cell Line | ER | PR | HER2 | CD44^pos^CD24^pos^ | CD44^pos^CD24^neg^ | CD44^neg^CD24^pos^ | CD44^neg^CD24^neg^ |
| --- | --- | --- | --- | --- | --- | --- | --- |

| AU565 | - | - | + | 0 | 61.7 | 38.3 | 0 |
| --- | --- | --- | --- | --- | --- | --- | --- |
| BT20 | - | - | - | 9.8 | 88.0 | 1.2 | 0.9 |
| BT483 | + | + | + | 0 | 13.1 | 86.8 | 0.1 |
| HS578T | - | - | +/- | 92.6 | 4.8 | 2.2 | 0.3 |
| MCF10 Ca1a | - | - | - | 89.9 | 9.8 | 0.2 | 0.1 |
| MCF7 | + | + | - | 61.6 | 0.4 | 35.0 | 3.0 |
| MDA MB 231 | - | - | + | 4.5 | 95.5 | 0 | 0 |
| MDA MB 435 | - | - | + | 95.4 | 3.8 | 0.1 | 0.8 |
| SK-BR3 | - | - | + | 1.6 | 34.7 | 62.8 | 0.8 |
| SUM1315mo | - | - | + | 93.2 | 6.2 | 0.5 | 0.1 |
| SUM149 | - | - | - | 93.1 | 6.8 | 0 | 0 |
| SUM159 | - | - | + | 96.1 | 3.0 | 0.8 | 0.1 |
| SUM185 | - | - | + | 0.1 | 1.5 | 98.3 | 0.1 |
| SUM229 | - | - | - | 19.3 | 78.9 | 1.3 | 0.4 |
| SUM52 | - | - | + | 0 | 0.8 | 99.1 | 0.1 |
| T47D | + | + | - | 0 | 37.8 | 61.7 | 0.4 |
| UACC-812 | + | + | + | 0 | 13.8 | 86.1 | 0.1 |
| UACC-893 | - | - | + | 0 | 12.9 | 87.1 | 0 |
| ZR75.1 | + | + | + | 62.9 | 0.1 | 36.9 | 0.2 |
