# Supplementary material for: Dynamic regulation of CD24 and the invasive, CD44posCD24neg phenotype in breast cancer cell lines
Source: Breast Cancer Res. 2009 Nov 11;11(6):R82. doi: 10.1186/bcr2449 (PMC2815544; doi:10.1186/bcr2449)
Supplement: Additional data file 4 — A table containing the CD44/CD24 expression profile of clones derived from a single CD44posCD24pos or CD44posCD24neg cell. [file bcr2449-S4.PPT]

## Slide 1
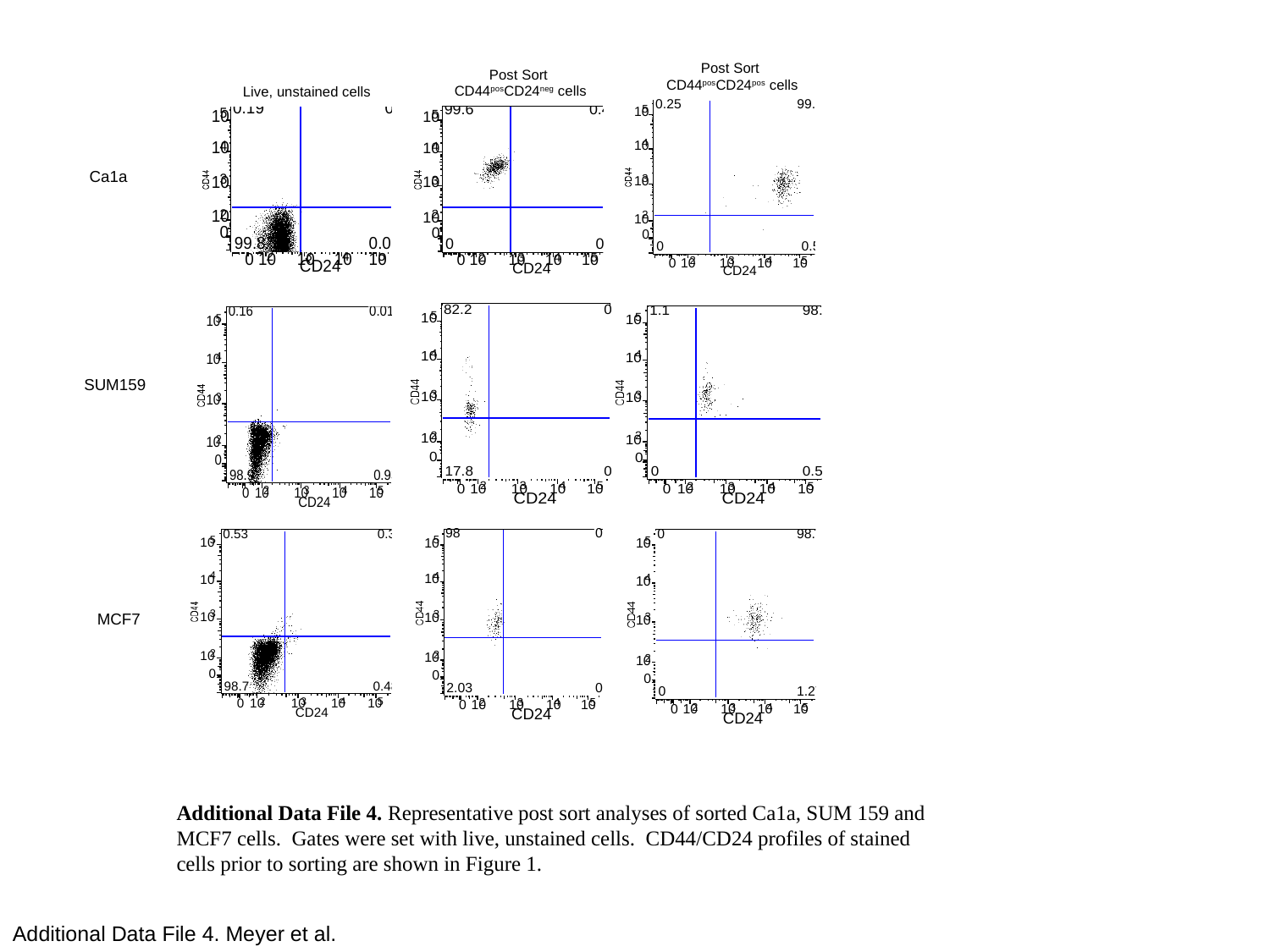

Post Sort
 CD44posCD24pos cells
Post Sort
 CD44posCD24neg cells
Live, unstained cells
Ca1a
SUM159
MCF7
Additional Data File 4. Representative post sort analyses of sorted Ca1a, SUM 159 and MCF7 cells. Gates were set with live, unstained cells. CD44/CD24 profiles of stained cells prior to sorting are shown in Figure 1.
Additional Data File 4. Meyer et al.
