# Supplementary material for: Dynamic regulation of CD24 and the invasive, CD44posCD24neg phenotype in breast cancer cell lines
Source: Breast Cancer Res. 2009 Nov 11;11(6):R82. doi: 10.1186/bcr2449 (PMC2815544; doi:10.1186/bcr2449)
Supplement: Additional file 5 — A figure containing representative post sort analyses of sorted Ca1a, SUM 159 and MCF7 cells. [file bcr2449-S5.PPT]

## Slide 1
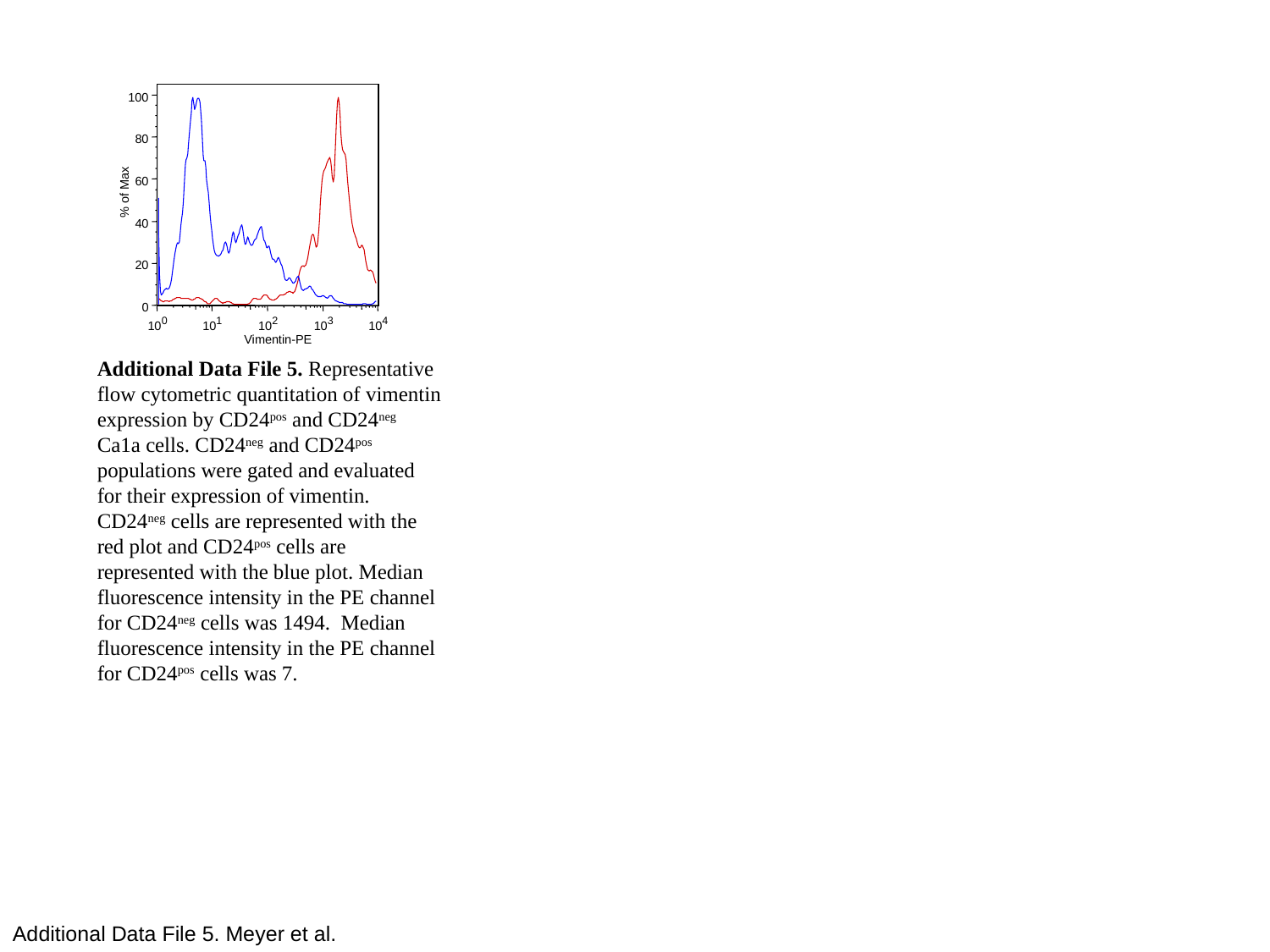

100
80
60
40
20
0
% of Max
0
1
2
3
4
10
10
10
10
10
Vimentin-PE
Additional Data File 5. Representative flow cytometric quantitation of vimentin expression by CD24pos and CD24neg Ca1a cells. CD24neg and CD24pos populations were gated and evaluated for their expression of vimentin. CD24neg cells are represented with the red plot and CD24pos cells are represented with the blue plot. Median fluorescence intensity in the PE channel for CD24neg cells was 1494. Median fluorescence intensity in the PE channel for CD24pos cells was 7.
Additional Data File 5. Meyer et al.
