# Supplementary material for: Dynamic regulation of CD24 and the invasive, CD44posCD24neg phenotype in breast cancer cell lines
Source: Breast Cancer Res. 2009 Nov 11;11(6):R82. doi: 10.1186/bcr2449 (PMC2815544; doi:10.1186/bcr2449)
Supplement: Additional file 6 — A figure containing representative flow cytometric quantitation of vimentin expression by CD44posCD24pos and CD44posCD24neg Ca1a cells. [file bcr2449-S6.DOC]

**Additional Data File 6. CD44/CD24 expression profile of clones derived from a single CD44^pos^CD24^pos^ or CD44^pos^CD24^neg^ cell.**

|  | Percentage of population | | | |
| --- | --- | --- | --- | --- |
|  | CD44^pos^CD24^pos^ | CD44^pos^CD24^neg^ | CD44^neg^CD24^pos^ | CD44^neg^CD24^neg^ |
| Clones initiated with CD44^pos^CD24^pos^ cells | | | | |
| Clone 1.1 | 99.7 | 0.3 | 0 | 0 |
| Clone 1.2 | 89.4 | 10.3 | 0.3 | 0 |
| Clone 1.3 | 82.7 | 17.3 | 0 | 0 |
| Clone 1.4 | 81.2 | 17.5 | 1.2 | 0.1 |
| Clone 1.5 | 91.9 | 8.1 | 0 | 0 |
| Clone 1.6 | 96.0 | 2.8 | 0.9 | 0.3 |
| Clone 1.7 | 97.7 | 2.0 | 0.3 | 0 |
| Clones initiated with CD44^pos^CD24^neg^ cells | | | | |
| Clone 2.1 | 91.9 | 6.6 | 1.4 | 0.1 |
| Clone 2.2 | 57.6 | 42.4 | 0 | 0 |
| Clone 2.3 | 80.7 | 19.2 | 0.1 | 0 |
| Clone 2.4 | 53.9 | 46.1 | 0 | 0 |
| Clone 2.5 | 84.2 | 15.6 | 0.2 | 0 |
